# Reduction in within-litter variation of piglet birth weight through dietary supplementation of 3-hydroxyisobutyric acid in sows

**DOI:** 10.3389/fvets.2025.1646332

**Published:** 2025-08-15

**Authors:** Long Che, Lizhu Niu, Le Liu, Mengyun Li, Wenying Huo, Hongyu Deng, Wen Chen, Lifeng Pang, Mengmeng Xu

**Affiliations:** ^1^College of Animal Science and Technology, Henan University of Animal Husbandry and Economy, Zhengzhou, China; ^2^College of Animal Science and Technology, Henan Agricultural University, Longzi Lake University Campus, Zhengzhou, China

**Keywords:** sow, gestation, piglet birth weight, 3-hydroxyisobutyric acid, lipid metabolism

## Abstract

Within-litter variations in birth weight (BW) influence piglet postnatal growth and survival rate. The present study investigated the effects of the valine metabolite 3-hydroxyisobutyric acid (3-HIB) on the birth weight of piglets by supplementing the sow diet with 3-HIB during late pregnancy. Forty sows were assigned randomly to the control (Con) group and 3-HIB supplementation groups (15 mg/kg body weight), with 20 sows per group. The experiment was conducted from day 85 of pregnancy until farrowing. 3-HIB supplementation significantly decreased the number of piglets with body weight < 1 kg, litter weight variation, and stillbirth number (*p* < 0.05) but had no significant effect on the organ index of newborn piglets. Compared to the control group, 3-HIB supplementation significantly increased the concentration of triglycerides in the placental tissue of sows (*p* < 0.05). The levels of total monounsaturated fatty acids and partial polyunsaturated fatty acids (C20:4 n-6, C20:5 n-3, and C22:6) in the plasma of piglets were significantly higher in the 3-HIB supplementation group than in the control group (*p* < 0.05). The results of lipid metabolism-related protein expression indicated that compared to the control group, the 3-HIB group significantly increased the expression of lipid transport-related proteins (solute carrier family 27a (SLC27A1) and fatty acid binding protein 3 [FABP3]) in the placenta of sows and fatty acid oxidation protein (carnitine palmitoyltransferase 1 [CPT-1]) in the muscle of piglets (*p* < 0.05). In conclusion, adding 3-HIB to the sow diet enhances piglet energy supply by promoting maternal-to-fetal fatty acid transport and fatty acid oxidation in piglet muscles, ultimately reducing within-litter body weight variation in newborn piglets.

## 1 Introduction

Reproductive performance in sows has improved through continuous genetic breeding and nutritional regulation. However, an increase in sow litter size leads to greater within-litter birth weight (BW) variation, which is an important factor to consider in feeding management (1, 2). This variation is also a key economic indicator; higher variation typically results in more low-birth-weight piglets, which increases the rates of culling and mortality (3). Within-litter birth weight variation is positively correlated with weaning weight and market weight in pigs. Piglets with higher birth weights usually have greater body weights at weaning because of their enhanced vitality and superior lactation ability (4), which facilitates faster growth and greater market weight (5). This variation in birth weight may be affected by genetic factors (6), litter size (7), maternal nutritional levels (8), and placental efficiency (9). The placenta is the only organ involved in the exchange of nutrients, metabolic waste, and respiratory gasses between sows and fetuses. The nutritional status of sows during pregnancy and the efficiency of placental nutrient transport are currently considered the main reasons for variation in piglet birth weight (9). Previous studies have shown that appropriately increasing the energy level in sow diets from 12.56 MJ of DE/kg to 14.23 MJ of DE/kg can improve lipid metabolism and nutrient transport in the placenta, thereby reducing variations in piglet birth weight (10). Therefore, improving the efficiency of nutrient transport in sow placentas via nutrition could be an effective strategy for reducing variation in piglet birth weight.

3-hydroxyisobutyric acid (3-HIB) is an intermediate valine metabolite (11). Studies in mice have shown that 3-HIB may be a vital lipid metabolism regulator that promotes cellular absorption of fatty acids by regulating the expression of fatty acid transport-related proteins (12, 13). A previous systematic investigation explored the effects of valine and 3-HIB on lipid metabolism in porcine mammary and intestinal epithelial cells. 3-HIB increased fatty acid intake by promoting the expression of fatty acid transporters in cells (14) and increasing cellular energy metabolism and adenosine triphosphate (ATP) production (15), ultimately promoting cell proliferation. Regulation of fatty acid transport by 3-HIB has been widely confirmed in energy metabolism in rats (16) and hepatic lipid metabolism in humans (17). However, studies on the role of 3-HIB in nutrient transport in the placenta of sows have not yet been conducted. Therefore, the objective of this study was to investigate the effects of supplementing sow diets with 3-HIB on the birth weight of newborn piglets and fatty acid transport efficiency in the sow placenta, thereby providing a basis for improving within-litter birth weight variation in piglets.

## 2 Materials and methods

### 2.1 Animal ethics statement

All experimental procedures complied with current animal protection laws (Ethics Approval Code: HNUAHE ER 2223101) and were approved by the Animal Care and Use Committee of Henan University of Animal Husbandry and Economy, in accordance with the Guide for the Care and Use of Laboratory Animals.

### 2.2 Experimental design and feeding management

This study was conducted at Pingdu Huayu Pig Breeding Technology R&D Co., Ltd., Qingdao City, Shandong Province. Forty healthy sows (Large White, on day 85 of gestation) with a parity level of 1–2 were used in this experiment. At 0–85 days of gestation, all sows were fed the same commercial diet with a digestive energy level of 3.1 Mcal/kg and a protein level of 12.8%. On day 85 of pregnancy, the sows were assigned to two treatment groups based on the principle of similar body weight and backfat thickness in a completely randomized design, with 20 replicates in each treatment group and one sow in each replicate. The sows in the control group (Con) were fed a basal diet (dietary formulations and nutritional compositions are shown in Table 1), and the sows in the treatment group (3-HIB) were supplemented with 15 mg/kg body weight of 3-HIB daily based on either the initial or post-parturition body weight in the basal diet. Sows were fed a diet mixed with 3-HIB at a dose corresponding to their body weight at each meal. 3-HIB, a valine metabolite, has not been reported in pig experiments. Therefore, the amount of 3-HIB in the diet was measured according to the addition of leucine metabolite β-hydroxy β-methylbutyrate (HMB) in sows, according to previous studies (18, 19). Each sow was fed twice daily (07:00 and 18:00 h), with a total daily intake of 2.8 kg/day until farrowing. The basal diet during late gestation was formulated to meet nutrient requirements as recommended by the National Research Council in 2012 (NRC 2012) (20), with all nutrient levels meeting or exceeding the NRC (2012) recommendations. All sows were housed in individual crates with an autoloading feeding system, and water was available *ad libitum*.

**Table 1:** Ingredients and nutrient content of diets (as-fed basis).

Ingredients	Proportion, %
Corn	59.14
Soybean meal	21.00
Wheat bran	15.00
Soybean oil	1.00
Limestone	1.10
Dicalcium phosphate	1.20
Salt	0.40
Choline chloride	0.15
L-Lys·H_2_SO_4_ (70%)	0.32
DL-Met (99%)	0.03
L-Thr (99%)	0.16
Vitamin and mineral premix^a^	0.50
Chemical composition^b^	
Crude protein, %	16.94
Digestible energy, Mcal/kg	3.20
Ca, %	0.77
Total phosphorus, %	0.64
Available phosphorus, %	0.35
Standardized total tract digestible phosphorus,%	0.37
Standardized ileal digestible Lys, %	0.87
Standardized ileal digestible Met, %	0.26
Standardized ileal digestible Thr, %	0.68

a Provided per kg of diet: vitamin A, 9,000 IU; vitamin D3, 1,000 IU; vitamin E, 86.2 IU; vitamin K3, 1.5 mg; vitamin B_1_, 1.0 mg; vitamin B_2_, 4.75 mg; vitamin B_6_, 3.5 mg; vitamin B12, 20 μg; niacin, 20 mg; pantothenic acid, 20 mg; folic acid, 0.85. Copper (CuSO_4_), 15 mg; iron (FeSO_4_), 100 mg; zinc (ZnSO_4_), 110 mg; manganese (MnSO_4_), 30 mg; selenium (Na_2_SeO_3_), 0.2 mg; iodine (CaI_2_O_6_), 0.3 mg.

b Calculated values.

### 2.3 Data and tissue sample collection

During the delivery of sows, the birth weight of each piglet was recorded to calculate litter weight. Litter size, number of live births, and number of stillbirths were recorded, and the weight coefficient of variation (CV) of each litter was calculated. Approximately 5 mL of blood was collected from the auricular marginal vein of sows (*n* = 12) and centrifuged (3,000 g for 15 min at 4°C) after standing for 15 min to obtain plasma samples; all samples were stored at −20°C for further examination. Three placental samples were collected from six sows in each treatment group, corresponding to the average piglet weight. The three collected placental tissue samples were analyzed for lipid metabolism-related indicators, lipid metabolism-related proteins, morphology, and immunofluorescence.

When sows farrowed, marks were made on the umbilical cords at both the sow and piglet ends to determine the placental sites corresponding to the piglets scheduled for slaughter. Placental samples corresponding to the average piglet weight were collected from six sows in each treatment group. These placental tissue samples were analyzed for lipid metabolism-related indicators—including triglycerides (TG), total cholesterol (TC), high-density lipoprotein cholesterol (HDL-C), low-density lipoprotein cholesterol (LDL-C), glucose (Glu)—as well as lipid metabolism-related proteins such as sterol regulatory element binding protein 1 (SREBP1), fatty acid synthase (FASN), acetyl-CoA carboxylase (ACC), solute carrier family 27a (SLC27A1), and fatty acid binding protein 3 (FABP3). Additionally, tissue morphology and immunofluorescence (FABP3) were examined. Six female piglets with weights close to the average weight per treatment group were selected from different gilts for blood sample collection and slaughter at farrowing. Blood was collected via the anterior vena cava and separated to obtain plasma samples that were then stored at −20°C for biochemical and fatty acid analysis. The piglets were euthanized after being anesthetized with an intravenous injection of Zoletil 50 (0.1 mg/kg body weight). The weights of the heart, liver, spleen, intestine, and other organs of the piglets were recorded to calculate the organ index. The organ index is calculated as the ratio of the organ weight to the body weight of piglets. Longissimus dorsi muscle samples were collected corresponding to the last rib and stored at −80°C for determining the expression levels of proteins related to lipid metabolism.

### 2.4 Biomarker analyses of lipid metabolism in the placenta and plasma

Approximately 100 mg of placental tissue was weighed, 1 mL of tissue lysis solution and iron beads were added, and the mixture was ground by a grinder (JXFSTPRP-64, Jingxin, Shanghai, China). After centrifugation at 12,000 g for 15 min at 4°C, the supernatant was collected to determine lipid metabolism indices. The concentrations of Glu, TG, TC, HDL-C, LDL-C, and total protein in the placenta and plasma were measured using commercial kits (Nanjing Jiancheng Bioengineering Institute, Nanjing, China), following the manufacturer’s instructions.

### 2.5 Composition of fatty acids in the plasma of piglets

The fatty acid composition of piglet plasma was determined by gas chromatography. First, fatty acids in the plasma were extracted using an organic solvent. The samples were then mixed and centrifuged to obtain an organic, aqueous, and protein phase. The organic phase was collected for the subsequent experiments. A methylating reagent was then added to the organic phase to convert the fatty acids into fatty acid methyl esters. After cooling to room temperature, the solution was treated with a saturated NaCl solution and n-hexane to obtain the n-hexane phase, which was used for instrument detection. The flow rate of the gas chromatograph was set to 1.5 mL/min. The capillary column dimensions were 30 m × 320 μm × 0.25 μm. The temperature of the injection port was gradually raised to 230°C within 2 min. Finally, because different fatty acid methyl esters formed different chromatographic peaks, a normalization method was used to calculate the area of each chromatographic peak, thereby reflecting the fatty acid content.

### 2.6 Immunofluorescent detection of proteins related to lipid metabolism

After fixing the tissue with 4% paraformaldehyde, the sample was cut into 5-μm sections using a microtome and placed on slides for drying. Subsequently, the slides were fixed using 4% paraformaldehyde for 30 min. The glass slides were washed 3 times for 5 min with phosphate-buffered saline (PBS). The slices were treated with a 0.1% Triton X-100 solution for 15 min. After washing with PBS 3 times, the slices were blocked with 5% bovine serum albumin (BSA) for 60 min. Subsequently, the slides were incubated with the FABP3 antibody solution and placed in a humidified chamber for incubation overnight at 4°C. Thereafter, the slides were incubated with a fluorescent-labeled secondary antibody at room temperature (25°C) for 60 min. The slides were overlaid with 4′,6-diamidino-2-phenylindole (DAPI) reagent, incubated in the dark for 10 min, and washed 3 times with PBS. Images were acquired using a fluorescence microscope (NIS-Elements, Nikon, Japan).

### 2.7 Western blotting

The expression of lipid metabolism-related proteins in the sow placenta (*n* = 6) and piglet muscle (*n* = 6) was detected. Samples from two sows or piglets were randomly combined into one sample, and three samples were obtained from each treatment group for Western blot analysis. The tissue samples were treated with lysates and then lysed using a grinder. The sample was then centrifuged at 12,000 g for 15 min at 4°C. The supernatant was collected, and the protein concentration of each sample was determined using a bicinchoninic acid (BCA) protein concentration assay kit (Thermo Scientific, MA, United States). For electrophoresis, 50 μg of sample was loaded per well/well. The electrophoresis time, membrane transfer time, antibody incubation time, and other parameters involved in the follow-up test process were as described in a previous study. Anti-phosphor-mammalian target of rapamycin (mTOR), anti-phospho-P70, anti-phospho-4EBP1, anti-SREBP1, anti-long-chain acyl-CoA synthetase (ACSL), anti-carnitine palmitoyltransferase (anti-CPT), and anti-carbamoyl-phosphate synthetase (anti-CAD) antibodies were purchased from Proteintech (Proteintech Group, Wuhan, China). Anti-CD36 and anti-ACC antibodies were purchased from Cell Signaling Technology (Danvers, MA, United States). Anti-FASN, anti-SLC27A1, and anti-FABP3 antibodies were purchased from Abcam (Cambridge, UK). Anti-rabbit IgG, anti-mouse IgG, and anti-β-actin antibodies were purchased from AmyJet Scientific (Wuhan, China). Protein expression was measured using the ImageJ software and normalized to β-actin expression levels.

### 2.8 Statistical analysis

The results of the production performance and related biochemical indices were analyzed for the homogeneity of variance and then tested using Student’s *t*-test in SPSS version 19.0 (IBM Corporation, Chicago, IL, USA). All data are presented through tables and figures as means and standard error of the mean (SEM). A *p*-value of < 0.05 was considered statistically significant, whereas values of 0.05 ≤ *p* < 0.10 were considered a tendency.

## 3 Results

### 3.1 Effect of dietary 3-HIB supplementation on the weight and organ index of newborn piglets

Newborn piglets were divided into four groups according to their body weight (<1.0 kg, 1.0–1.2 kg, 1.2–1.4 kg, and >1.4 kg, respectively). The body weights of the pigs of different grades were statistically analyzed (Table 2). The addition of 3-HIB to the diet had no significant effect on the total number of offspring. 3-HIB supplementation significantly reduced the number of piglets below 1 kg compared to the Con group (*p* < 0.05), but had no significant effect on piglets of other body weight grades. Compared to the Con group, 3-HIB supplementation significantly reduced the number of stillbirths, the rate of weak piglets (*p* < 0.05), and the variation in birth weight of piglets (*p* < 0.05). 3-HIB supplementation to the sow diet did not affect the organ indices of newborn piglets (Table 3).

**Table 2:** The effect of dietary 3-HIB supplementation on the weight of newborn piglets.

Items	Con	3-HIB	SEM	*p*-value
Litter size	15.56	14.41	0.44	0.194
<1.0 kg	1.84	0.89	0.23	0.036
1.0–1.2 kg	2.47	2.16	0.29	0.590
1.2–1.4 kg	3.45	4.11	0.37	0.385
>1.4 kg	6.70	6.72	0.51	0.983
CV	0.20	0.16	0.01	0.006
Rate of weak piglets	0.12	0.06	0.02	0.048
Stillbirth	1.39	0.20	0.19	0.001

Con, control, basal diet (20 litters of piglets); 3-HIB, 3-hydroxyisobutyric acid, basal diet + 15 mg/kg birth weight (BW) of 3-HIB (20 litters of piglets). CV, within-litter birth weight coefficient of variation. SEM, standard error of means.

**Table 3:** The effect of dietary 3-HIB supplementation on the organ index of piglets (g/kg).

Items	Con	3-HIB	SEM	*p*-value
Heart index	6.52	6.47	0.22	0.912
Liver index	23.95	24.34	0.86	0.837
Lung index	15.35	16.10	0.57	0.543
Kidney index	7.63	7.41	0.28	0.716
Spleen index	0.84	0.86	0.03	0.778
Stomach index	4.97	4.84	0.19	0.746
Intestine index	37.73	37.33	1.20	0.881

Con, Control, basal diet (*n* = 6); 3-HIB, 3-hydroxyisobutyric acid, basal diet + 15 mg/kg birth weight (BW) of 3-HIB (*n* = 6). Organ index (g/kg): organ weight (g)/body weight (kg). SEM: standard error of means.

### 3.2 Effect of dietary 3-HIB supplementation on lipid metabolism in sow placenta and plasma

Lipid metabolism-related indicators in the plasma and placental homogenates of sows, including glucose (Glu), triglyceride (TG), total cholesterol (TC), high-density lipoprotein cholesterol (HDL-C), and low-density lipoprotein cholesterol (LDL-C), were determined (Table 4). Compared to the Con group, the addition of 3-HIB to the diet of sows had no significant effect on the concentration of each index in the plasma, but tended to increase the concentration of TG (*p* = 0.069). In the placenta of sows, TG concentrations were significantly higher in the 3-HIB supplementation group than in the Con group (*p* < 0.05). In addition, 3-HIB supplementation tended to increase the Glu concentration (*p* = 0.079).

**Table 4:** The effect of dietary 3-HIB supplementation on the plasma biochemicals of plasma and placenta.

Items	Con	3-HIB	SEM	*p*-value
Plasma				
Glu (mmol/L)	2.98	2.96	0.20	0.968
TG (mmol/L)	0.33	0.49	0.04	0.069
TC (mmol/L)	1.16	0.89	0.10	0.180
HDL-C (mmol/L)	0.69	0.73	0.04	0.630
LDL-C (mmol/L)	1.11	0.97	0.08	0.411
Placenta				
Glu (mmol/gprot)	0.63	0.70	0.02	0.079
TG (mmol/gprot)	0.20	0.25	0.02	0.039
TC (mmol/gprot)	0.35	0.38	0.02	0.520
HDL-C (mmol/gprot)	0.22	0.18	0.02	0.353
LDL-C (μmol/gprot)	26.68	28.11	1.81	0.704

Con, Control, basal diet (plasma: *n* = 12; placenta: *n* = 6); 3-HIB, hydroxyisobutyric acid, basal diet + 15 mg/kg BW of 3-HIB (plasma: *n* = 12; placenta: *n* = 6); Glu, glucose; TG, triglyceride; TC, total cholesterol; HDL-C, high-density lipoprotein cholesterol; LDL-C, low-density lipoprotein cholesterol; SEM, standard error of means.

### 3.3 Effect of dietary 3-HIB supplementation on the composition of fatty acids in the plasma of piglets

The fatty acid composition in the plasma of newborn piglets was detected (Table 5). For saturated fatty acids, higher proportions of C18:0 and total saturated fatty acids were observed in the 3-HIB group than in the Con group (*p* < 0.05). However, 3-HIB supplementation did not affect the monounsaturated fatty acid content in the plasma of piglets. For polyunsaturated fatty acids, C20:4 n-6, C20:5 n-3, C22:6, and total polyunsaturated fatty acid concentrations were significantly higher in the 3-HIB supplementation group than in the Con group (*p* < 0.05).

**Table 5:** The effect of dietary 3-HIB supplementation on the composition of fatty acids in the plasma of piglets.

Fatty acids	Con	3-HIB	SEM	*p*-value
Saturated fatty acids (μg/mL)				
C14:0	0.84	0.92	0.03	0.188
C15:0	0.35	0.43	0.02	0.060
C16:0	24.69	28.09	0.95	0.056
C17:0	0.79	1.02	0.06	0.078
C18:0	18.94	22.19	0.89	0.047
Total	45.60	52.65	1.92	0.045
Monounsaturated fatty acids (μg/mL)				
C16:1	2.98	3.13	0.08	0.424
C18:1 n-9	20.13	21.21	0.50	0.339
C20:1	0.32	0.34	0.01	0.247
Total	23.44	24.69	0.59	0.344
Polyunsaturated fatty acids (μg/mL)				
C18:2 n-6	6.38	7.49	0.35	0.109
C20:2	0.90	1.00	0.05	0.391
C20:3 n-6	0.91	0.95	0.02	0.253
C20:4 n-6	21.09	26.83	1.49	0.028
C20:5 n-3	0.29	0.37	0.02	0.021
C22:6	3.83	4.51	0.18	0.025
Total	33.40	41.17	2.01	0.027

Con, Control, basal diet (*n* = 4); 3-HIB, hydroxyisobutyric acid, basal diet + 15 mg/kg birth weight (BW) of 3-HIB (*n* = 4). SEM, standard error of means.

### 3.4 Effect of dietary 3-HIB supplementation on the expression of lipid metabolism-related proteins in the placenta of sows

**Figure 1:**
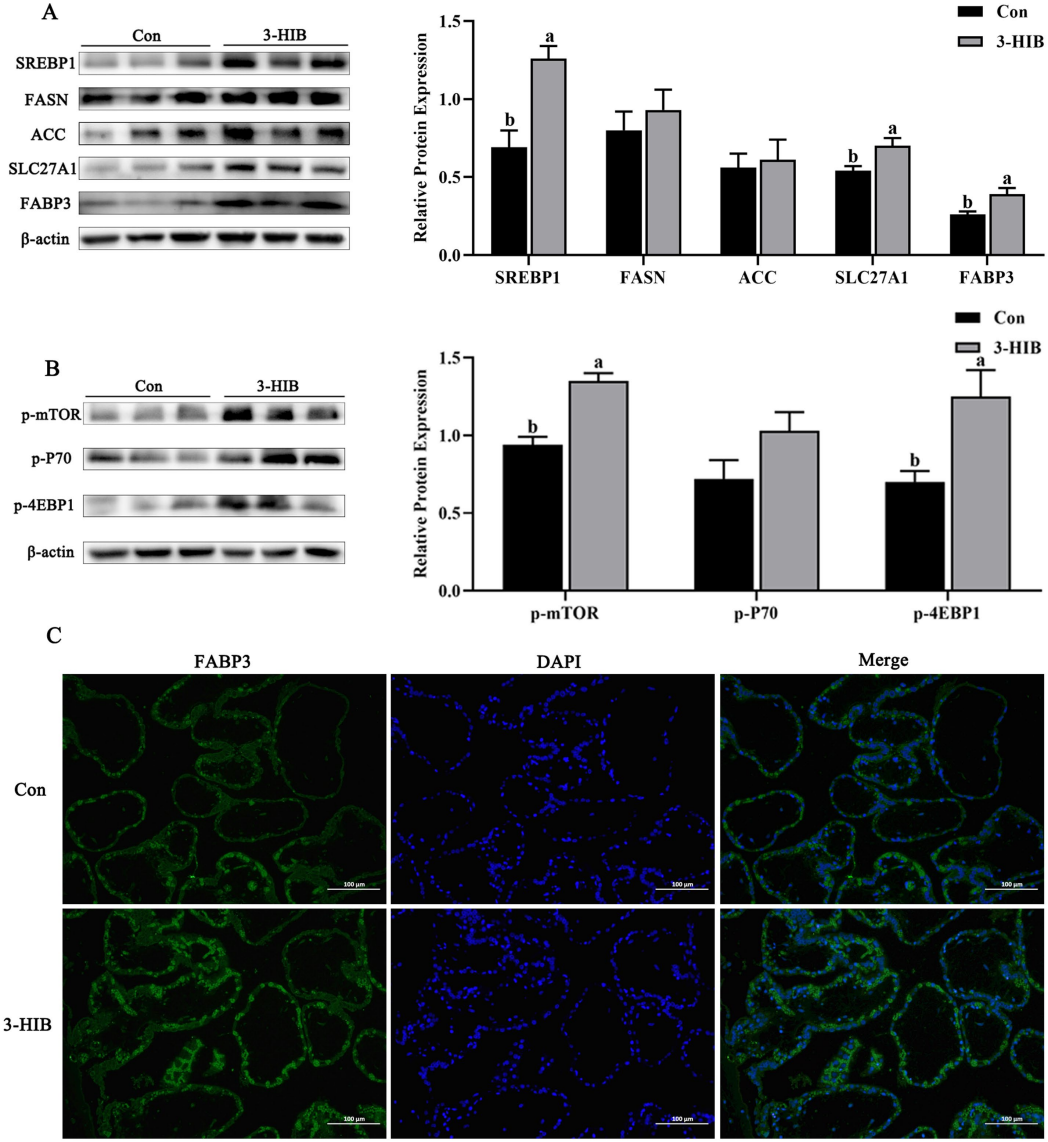
The effect of dietary 3-HIB supplementation on the expression of lipid metabolism-related proteins in the placenta. **(A)** Expression levels of lipid metabolism-related proteins in the placenta of sows (*n* = 6). SREBP1, sterol regulatory element binding protein 1; FASN, fatty acid synthase; ACC, acetyl-CoA carboxylase; SLC27A1, solute carrier family 27a; FABP3, fatty acid binding protein 3. **(B)** Phosphorylation level of the mTOR signaling pathway in the placenta of sows (*n* = 6). p-mTOR, phosphorylated mTOR; p-P70, phosphorylated P70; p-4EBP1, phosphorylated 4EBP1. **(C)** Immunofluorescent staining of FABP3 was performed in the placenta of sows (*n* = 6). DAPI: images of cell nuclei stained with Hoechst 33342; Merge: the merged image of green fluorescence and blue nuclei. Data represent mean ± standard error of mean; means not sharing the same letter are significantly different (*p* < 0.05).

To investigate the reasons for the increase in plasma triglycerides and fatty acids in newborn piglets in the 3-HIB supplementation group, this study further analyzed the expression of lipid metabolism-related proteins in the placenta (Figure 1). Compared to the Con group, the 3-HIB addition group showed significantly increased expression of SREBP1 protein in the placenta (*p* < 0.05), along with significantly upregulated expression of its downstream fatty acid transport-related proteins, including SLC27A1 and FABP3 (*p* < 0.05, Figure 1A). The results of placental tissue immunofluorescence analysis were consistent with those of western blotting. The 3-HIB supplementation group showed higher fluorescence intensity of the FABP3 protein (Figure 1C). The expression of *de novo* fatty acid synthesis-related proteins (FASN and ACC) was not affected by 3-HIB supplementation (Figure 1A). Furthermore, assessment of the expression of mTOR signaling pathway-related proteins revealed that p-mTOR and p-4EBP1 were significantly increased in the 3-HIB addition group compared to the Con group (*p* < 0.05). These findings suggest that 3-HIB regulates the expression of downstream fatty acid transporters by activating the mTOR signaling pathway.

### 3.5 Effect of dietary 3-HIB supplementation on the expression of lipid metabolism-related proteins in the muscle of piglets

**Figure 2:**
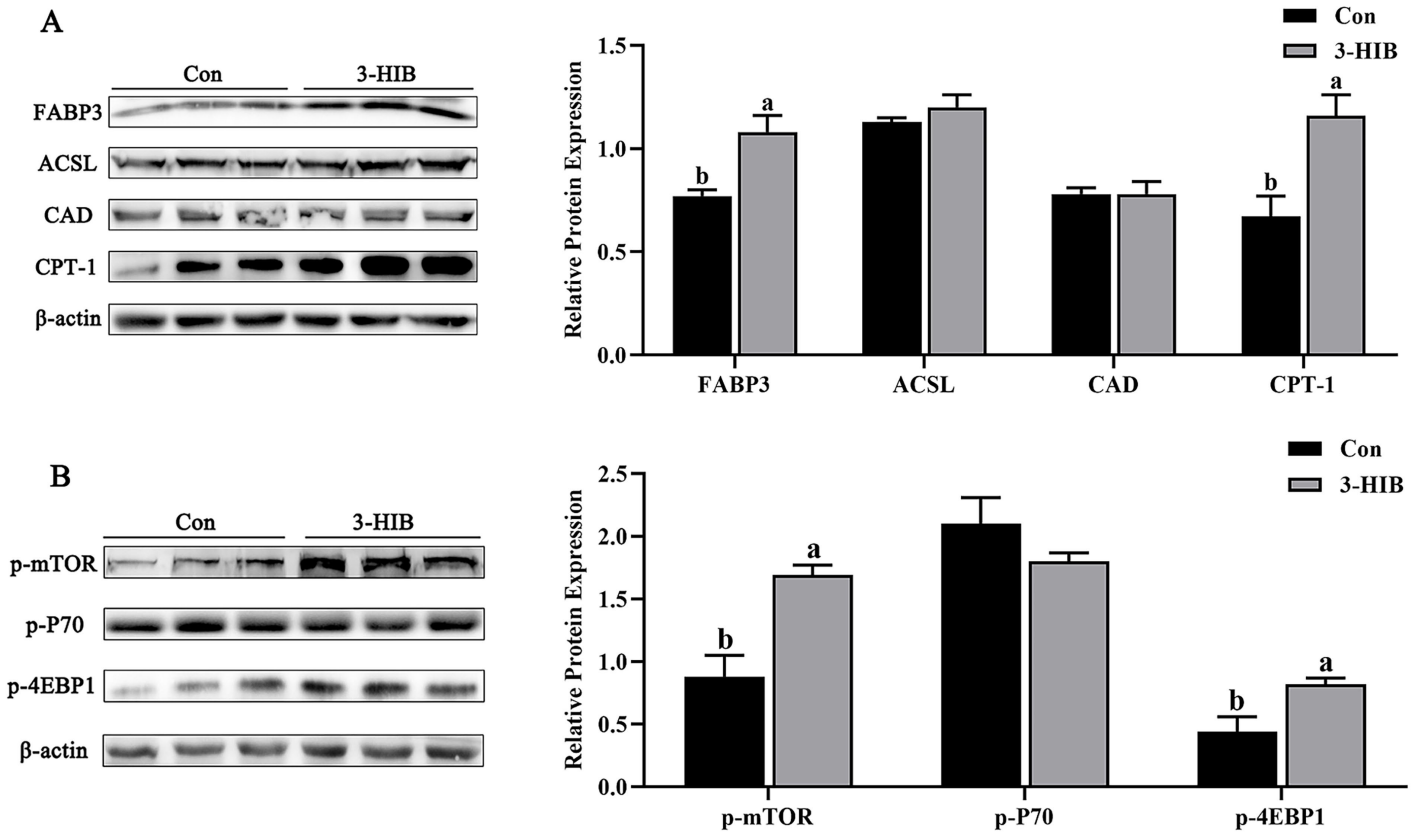
The effect of dietary 3-HIB supplementation on the expression of lipid metabolism-related proteins in the muscle of piglets. **(A)** Expression levels of lipid metabolism-related proteins in the muscle of piglets (*n* = 6). FABP3, fatty acid binding protein 3; ACSL, long-chain acyl-CoA synthetase; CAD, Carbamoyl-phosphate synthetase 2, Aspartate transcarbamylase, and Dihydroorotase; CPT-1, Carnitine Palmitoyltransferase 1. **(B)** Phosphorylation level of the mTOR signaling pathway in muscle of piglets (*n* = 6). p-mTOR, phosphorylated mTOR; p-P70, phosphorylated P70; p-4EBP1, phosphorylated 4EBP1. Data represent mean ± standard error of mean; means not sharing the same letter are significantly different (*p* < 0.05).

The present study investigated the expression of lipid metabolism-related proteins in piglet muscle tissues (Figure 2). The expression of FABP3 protein in muscle tissue was significantly higher in the 3-HIB addition group than in the Con group (*p* < 0.05). Simultaneously, the expression of the rate-limiting enzyme of fatty acid oxidation (carnitine palmitoyltransferase 1 [CPT-1]) in the muscle tissue was significantly increased in the 3-HIB addition group (*p* < 0.05), indicating that increased fatty acid oxidation may provide more energy for cell proliferation. The expression of mTOR signaling pathway-related proteins in the muscle tissue was consistent with that in the placenta. The expression of p-mTOR and p-4EBP1 proteins was significantly higher in the 3-HIB addition group than in the Con group (*p* < 0.05).

## 4 Discussion

The birth weight of piglets is closely related to their growth and survival rates in later stages (21). Previous studies have shown that 30% of piglets with a birth weight < 1 kg do not survive weaning, whereas the rate is even lower (47%) in piglets with a birth weight < 750 g (3). Therefore, improving piglet birth weight and litter uniformity is key to improving production efficiency. Maternal nutrition is the most critical factor affecting within-litter birth weight variation in piglets (22). As a unique nutrient-providing organ for piglets before birth, the nutrient transport efficiency of the placenta is vital for fetal porcine development and piglet birth weight (23). Previous studies have shown that the concentrations of glucose and some essential amino acids in the umbilical vein blood of piglets with intrauterine growth restriction (IUGR) are significantly reduced compared with those in normal newborn piglets (24), indicating that the nutrient transport efficiency of the placenta is the main factor affecting the birth weight of piglets. Sows have a diffuse placenta, with fetuses diffusely distributed along the uterine horns on both sides (25). Our previous study reported that fetal pig weight was significantly higher at the end of the uterine horn (near the ovary) than at the beginning of the uterine horn (near the uterine body), indicating that the difference in placental nutrient transport efficiency at different sites may be a major reason for the variation in piglet birth weight (10). Balanced nutritional distribution in sow placental lipid and energy metabolism is an effective way to reduce variation in piglet birth weight (10, 26). This study found that dietary supplementation of 3-HIB in sows during late gestation significantly reduced birth weight variation among piglets. The improved uniformity in within-litter birth weight may be attributed to 3-HIB’s role in enhancing placental fatty acid transport. Specifically, the 3-HIB group exhibited significantly higher plasma concentrations of certain fatty acids in piglets, suggesting enhanced fetal nutritional reserves.

Valine is a branched-chain amino acid involved in energy metabolism and immune regulation, in addition to participating in milk protein synthesis (11). A previous study reported that adding valine to the diet of lactating sows increased milk fat content and piglet weaning weight significantly (27), indicating that valine participates in the regulation of lipid metabolism in sow mammary gland epithelial cells (15). Studies have also shown that 3-HIB, an intermediate product of valine metabolism, is a key mediator of lipid metabolism in mammary epithelial cells. Furthermore, 3-HIB is the only substance that escapes mitochondrial oxidation and is secreted into the cytoplasm to participate in fatty acid transport during valine metabolism in mice (12, 13). In late gestation, the fetus’s demand for nutrients increases significantly because it is the main period of weight gain (28). The addition of 3-HIB to the diet of sows significantly increased the levels of fatty acids in the blood of piglets, thereby improving variation in birth weight. Previous studies have demonstrated that 3-HIB supplementation in mouse drinking water significantly elevated 3-HIB concentration in skeletal muscle tissues compared to that in the control group, indicating that 3-HIB is absorbed by intestinal cells, enters the bloodstream, and is transported to target organs (12). Therefore, the increase in fatty acid concentration in piglet blood may be attributed to 3-HIB-mediated promotion of fatty acid transport in the placenta (29). As an important transcriptional regulator of lipid metabolism, sterol regulatory element binding protein 1 (SREBP1) is activated and translocated from the cytoplasm to the nucleus to regulate the expression of target genes involved in fatty acid metabolism and promote fatty acid transport (30). Activated SREBP1 triggers the expression of downstream fatty acid transporters (e.g., SLC27A1 or FABP3) to facilitate fatty acid transport, thereby providing substrates for triglyceride synthesis or fatty acid oxidation (31). The present study found that the significant increase in the expression of fatty acid transport-related proteins (SREBP1, SLC27A1, and FABP3) in the placenta of sows in the 3-HIB group may be the primary reason for the increase in blood fatty acid concentration in piglets. The elevated placental TG concentration in 3-HIB-supplemented sows likely reflects enhanced fatty acid transport. No significant differences in maternal plasma lipid metabolism indices were observed between groups. We hypothesized that this stability may reflect systemic homeostatic regulation, whereby sows maintain consistent blood lipid levels through hepatic or adipose tissue compensatory mechanisms. Previous studies have demonstrated that upregulating fatty acid transporters in porcine placental trophoblast cells enhanced long-chain fatty acid transport, thereby promoting fetal and placental growth (32), which supports the results of this study. On the other hand, the organ index of piglets can partially reflect their organ metabolic function and growth status. In this study, we measured organ index in newborn piglets. Since these piglets had not yet been exposed to external nutrients (e.g., milk or feed) and the experimental duration was short, these factors may explain the absence of significant changes in organ index.

Mammalian target of rapamycin (mTOR) is a key kinase involved in the regulation of cell growth, protein synthesis, fat synthesis, and numerous other physiological processes (33). Studies have shown that treatment of cells with the mTOR signaling inhibitor rapamycin significantly reduces insulin-induced activation of SREBP1 and cellular lipid synthesis, confirming that mTOR plays an important role in insulin-induced SREBP1 activation and lipid metabolism (34). The mTOR signaling pathway mediates SREBP1, which is involved in the regulation of cellular lipid synthesis in porcine mammary epithelial cells (35). The addition of 3-HIB to the diet of sows increased mTOR phosphorylation significantly, which in turn promoted SREBP1 protein expression. The finding is consistent with our previous results, which showed that an appropriate concentration of 3-HIB promotes cell lipid metabolism and proliferation by activating the mTOR signaling pathway (15). Fatty acid β-oxidation is the primary pathway via which cells obtain energy through mitochondrial metabolism of fatty acids, supporting piglet growth (36). During the process, fatty acids undergo activation, are translocated to the mitochondria, and undergo oxidation cycles. Carnitine palmitoyltransferase I (CPT1) is a key rate-limiting enzyme in fatty acid β-oxidation, playing a regulatory role (37). The results of the present study showed that the mTOR signaling pathway was activated and CPT1 protein expression was promoted in the muscle tissue of piglets in the 3-HIB treatment group, which may have provided a higher energy supply for piglets. Similar studies have confirmed that the addition of lipids to the diet of piglets significantly increases intestinal lipid metabolism and ATP synthesis (38). In summary, the present study established that 3-HIB can activate the mTOR signaling pathway to promote the expression of lipid metabolism-related proteins, providing a theoretical basis for supplementation of sow diets with 3-HIB to reduce within-litter birth weight variation in newborn piglets.

However, the lack of dose gradients in the present study makes it difficult to determine the optimal dosage and dose-dependent effects. The dosage of 3-HIB in this study was based on previous HMB research (a leucine metabolite). Although both 3-HIB and HMB are branched-chain amino acid metabolites, they differ structurally and are synthesized via distinct enzymatic pathways, likely with varying efficiencies. These results provided preliminary insights into 3-HIB’s effects on sow reproductive performance. In future research, multiple gradients of 3-HIB concentration should be set up to systematically explore the dose–response relationship. This would help improve nutritional regulation strategies and enhance practical applications in swine production. Carbohydrates and proteins, along with fats, are key factors influencing piglet growth. However, the effects of 3-HIB on carbohydrate and protein metabolism remain unexplored. Further research into how 3-HIB affects glucose and amino acid transport efficiency in sow placentas may enhance our understanding of its role in improving piglet birth weight. It is important to note that a limitation of this study was the use of a t-test, which did not account for litter effects (non-independence of piglets). Future studies should employ linear mixed models with sow as a random effect to achieve more accurate treatment effect estimation.

## 5 Conclusion

This study is the first to demonstrate that the valine metabolite 3-HIB can reduce within-litter birth weight variation in piglets by upregulating the expression of placental fatty acid transporters in sows and enhancing maternal-to-fetal fatty acid supply, thereby offering a nutritional intervention strategy for addressing poor litter uniformity in swine production.

## Data Availability

The original contributions presented in the study are included in the article/supplementary material, further inquiries can be directed to the corresponding author.
